# Misalignment between perceptual boundaries and weight categories reflects a new normal for body size perception

**DOI:** 10.1038/s41598-021-89533-5

**Published:** 2021-05-17

**Authors:** Annie W. Y. Chan, Danielle L. Noles, Nathan Utkov, Oguz Akbilgic, Webb Smith

**Affiliations:** 1 Division of Psychology, Department of Life Sciences, Centre for Cognitive Neuroscience, Brunel University London, Gaskell Building, Room 219, Uxbridge, UB8 3PH UK; 2 School of Medicine, University of Tennessee Health Science Center, Memphis, TN USA; 3 Department of Bioinformatics, University of Tennessee Health Science Center, Memphis, TN USA; 4 Department of Health Informatics and Data Science, Parkinson School of Health Informatics and Public Health, Loyola University Chicago, Maywood, IL USA; 5 Department of Pediatrics, University of Tennessee Health Science Center, Memphis, TN USA

**Keywords:** Human behaviour, Psychology, Public health, Weight management

## Abstract

Combatting the current global epidemic of obesity requires that people have a realistic understanding of what a healthy body size looks like. This is a particular issue in different population sub-groups, where there may be increased susceptibility to obesity-related diseases. Prior research has been unable to systematically assess body size judgement due to a lack of attention to gender and race; our study aimed to identify the contribution of these factors. Using a data-driven multi-variate decision tree approach, we varied the gender and race of image stimuli used, and included the same diversity among participants. We adopted a condition-rich categorization visual task and presented participants with 120 unique body images. We show that gender and weight categories of the stimuli affect accuracy of body size perception. The decision pattern reveals biases for male bodies, in which participants showed an increasing number of errors from leaner to bigger bodies, particularly under-estimation errors. Participants consistently mis-categorized overweight male bodies as normal weight, while accurately categorizing normal weight. Overweight male bodies are now perceived as part of an expanded normal: the perceptual boundary of normal weight has become wider than the recognized BMI category. For female bodies, another intriguing pattern emerged, in which participants consistently mis-categorized underweight bodies as normal, whilst still accurately categorizing normal female bodies. Underweight female bodies are now in an expanded normal, in opposite direction to that of males. Furthermore, an impact of race type and gender of participants was also observed. Our results demonstrate that perceptual weight categorization is multi-dimensional, such that categorization decisions can be driven by ultiple factors.

## Introduction

Body weight perception differs across cultures^1–3^, perceptual biases can vary due to social-economic status, religion, ethnicity, muscular tone, location, and/or gender, thus highlighting the diverse variations in body weight perception^4^. Much research has focused on weight estimation of oneself, especially how differences in ethnicity affect self-reporting of one’s own weight or their preferred weight. It has been suggested^1–3^ that African American women seem to prefer a larger body and may often perceive an overweight body as normal weight, and they may tend to under-estimate their own weight. Meanwhile, Caucasian American women prefer leaner bodies and may often perceive underweight as normal, and they are more likely to over-estimate their own weight. Others^5,6^ have observed that people, predominately Caucasian female participants, tend to under-estimate Caucasian bodies. Since multiple demographic factors (e.g. ethnicity and gender of the stimuli as well as of the participants) can contribute to weight perception biases, a data-driven multivariate approach using a machine learning, such as decision tree, is thus necessary, as it will allow identification of those critical factors that would otherwise be missed.

Visual perception of body weight has been investigated from visual, social, and health psychology perspectives. For example, whether gender or ethnicity influences body size preference. A recent questionnaire survey^7^ reported that African–American and Hispanic men preferred women with larger bodies, whereas Caucasian American men preferred women with thinner bodies. Others^1^ have reported that such racial disparity was stronger in women than in men. Researchers have also concentrated on self-perception of participants’ own body weight^4,8,9^. Although some recent studies have examined weight perception from the perspective of the observers, they have only recruited participants exclusively from one of the genders, mostly women^2,10–12^, presented body images of only one gender^5,12–14^, presented only Caucasian stimuli^5,15,16^ or used line drawings without colour information^17^. One recent report^12^ has examined weight estimation of both self and others using a very realistic set of stimuli, but with only female stimuli and female participants. Others have also attempted to illustrate the discrepancy of body weight across different ethnic groups^2,4,11,14,18^. Several vision scientists^19–25^ have adopted a well-controlled visual psychophysics approach, using visual adaptation to investigate the impact of context and exposure on body size and shape misperception. Despite these investigations, more work is needed in order to quantify and delineate the contribution of gender, ethnicity, and identity in parallel.

Our principle is that using a well-balanced psychophysics task with a wide variety of stimuli, it will allow us to measure accuracy and error rate, and it will also allow us to define the perceptual categorization patterns and biases. A psychophysics paradigm can also measure behaviour repeatedly with an expansive variety of conditions and stimuli. Some of the previous studies have used body images in their survey; unfortunately, these stimuli were primarily line drawing, silhouette, or schematic figures^26,27^, which failed to capture crucial details of a person such as skin colors, textures, and facial features^2,3,8,14,28^. Furthermore, many have tested only a limited number of stimuli, varying from 7 to 30 images^3,11,18,28,29^. A few recent studies^9,12,13^ have used a wider selection of stimuli, but have only presented female body images.

To address these gaps of knowledge, here we aim: (1) To identify key demographical features affecting visual perception of body weight and estimation of weight boundaries of others. (2) To increase our understanding of how variety affects weight categorization, with a broader spectrum of demographic features (e.g. gender, ethnicity) at both stimulus and participant levels. (3) To measure and compare perceptual boundaries and the recognized weight categories (Body Mass Index, BMI).

## Results

### Are people good at categorizing body size?

**Figure 1:**
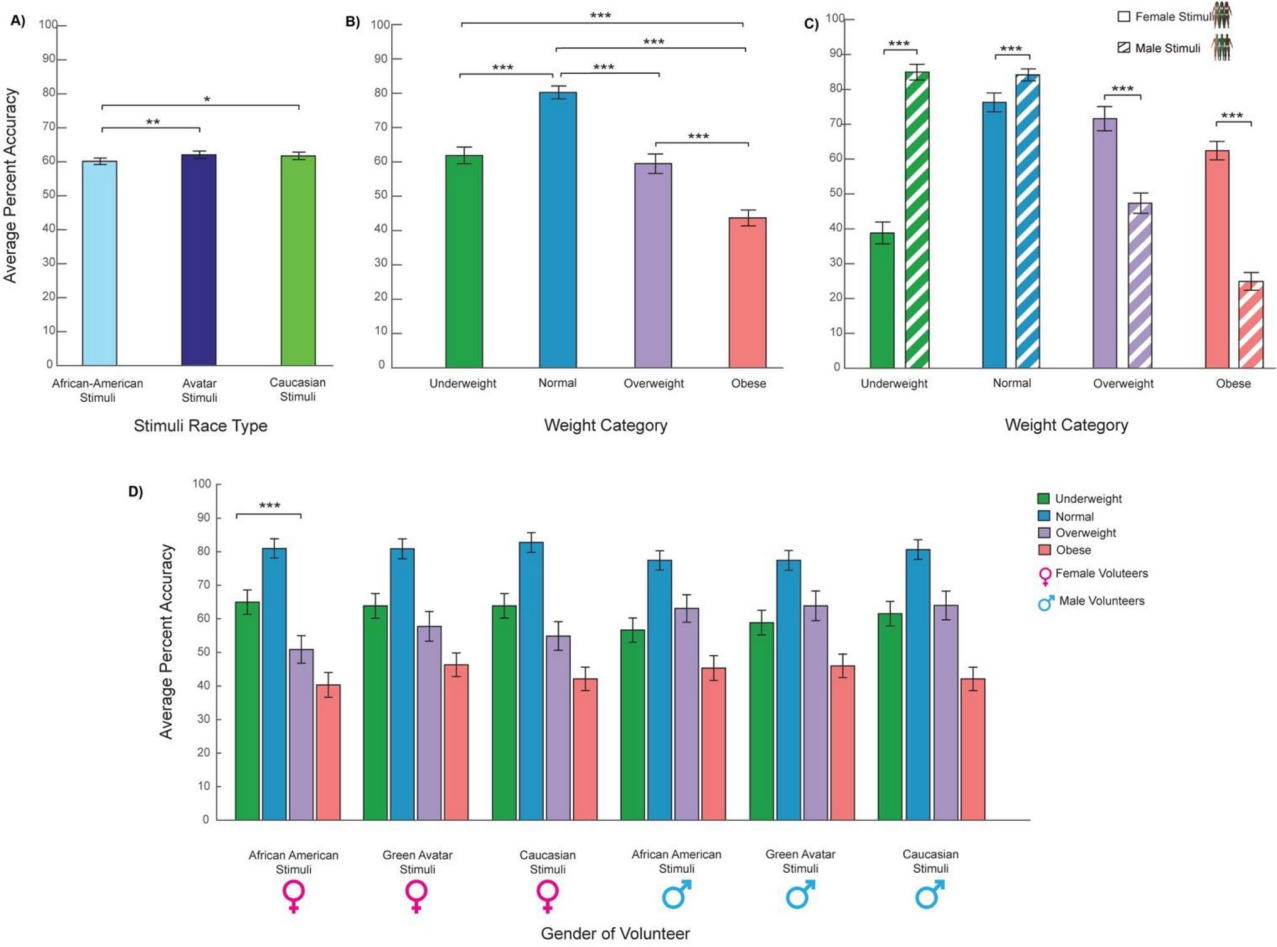
Performance in terms of average percent accuracy. (**A**) A significant main effect of stimuli race type: there was a small but significant difference across stimuli race type, whereby participants performed best for the Green Avatar stimuli. (**B**) A significant main effect of stimuli weight categories was found, whereby participants performed best for normal stimuli and worst for obese stimuli. (**C**) A significant interaction of weight category and stimuli gender: specifically, participants were more accurate when categorizing male (hatched bars) normal and underweight bodies relative to the corresponding female bodies, whereas they were more accurate in categorizing female overweight and obese bodies relative to male bodies. (**D**) Illustrating a significant three-way interaction of participants’ gender, stimuli race type and stimuli weight category. In particular, accuracy for underweight female African American bodies was significantly better than accuracy for overweight female African American bodies.

We have used percent accuracy as a measurement of performance (a univariate approach, Fig. 1). Participants were not at ceiling in terms of their performance, though most of them performed above chance level (25%). Overall, our participants did not demonstrate a uniform level of performance across all conditions; their performance was modulated by demographic factors such as the Race Types, Genders and/or Weight Categories of body stimuli they observed, as well as participants’ Gender.

#### Main effect of the stimulus race types

Participants did not perform equally across all stimulus race types; small but significant differences across race types (Fig. 1A) were observed. Specifically, a significant main effect of the Stimulus Race Type, *F*(2,112) = 4.37, *p* < 0.015, η_p_^2^ = 0.072 was found. Further tests showed that across all participants, categorization performance was best for Green Avatar (the control race type), followed by Caucasian, then African–American stimuli. Paired samples t-tests showed significant better performance for Caucasian > African–American (*t* = 2.19, *p* < 0.032); for Green Avatar > African American (*t* = 2.79, *p* < 0.007), but no significant difference between Green Avatar vs Caucasian (*t* = 0.58, *p* < 0.564).

#### Main effect of BMI weight categories

Responses were not the same for all weight categories (Fig. 1B), participants performed significantly better for Normal than Underweight and Overweight, and worse for Obese weight. This is confirmed by a significant main effect of BMI weight category, *F*(3,168) = 32.787, *p* < 0.001, η_p_^2^ = 0.369. Follow-up paired t-tests showed a significantly higher percent accuracy when categorizing Normal weight compared to other weight categories: Normal > Underweight (*t* = 6.478, *p* < 0.001), Normal > Overweight (*t* = 5.412, *p* < 0.001), Normal > Obese (*t* = 9.216, *p* < 0.001). There are also significant differences between Overweight > Obese (*t* = 4.994, *p* < 0.001) and Underweight > Obese (*t* = 4.695, *p* < 0.001). No significant difference was found between Underweight and Overweight (*t* = 0.592, *p* > 0.556).

#### Interaction between stimuli gender and stimuli weight categories

Intriguingly, participants were more accurate when categorizing leaner male stimuli (underweight and normal bodies) relative to those leaner female stimuli. On the other hand, participants were more accurate when categorizing heavier female stimuli (Overweight and Obese bodies) relative to those heavier male bodies (Fig. 1C). A significant two-way interaction between stimulus gender and stimulus weight category was found *F*(3,168) = 210.293, *p* < 0.001, η_p_^2^ = 0.790. Follow-up paired t-tests confirmed that significant difference between accuracy rates for female and male stimuli for each weight category: Underweight female < male stimuli (*t* = − 18.912, *p* < 0.001), Normal female < male stimuli (*t* = − 3.499, *p* < 0.001), Overweight female > male stimuli (*t* = 8.587, *p* < 0.001), Obese female > male stimuli (*t* = 15.732, *p* < 0.001).

#### Interaction between stimuli race type, stimuli weight category, and gender of participants

We have observed a significant 3-way interaction between stimuli race type, stimuli weight category, and gender of the participants (Fig. 1D), F(3,336) = 230.174, p < 0.026, η_p_^2^ = 0.042. In general, female participants were more accurate for Underweight than Overweight bodies, and in particular, these female participants were significantly more accurate for African American Underweight stimuli than African American Overweight stimuli (*t* = 2.962, *p* < 0.006). In contrast, male participants did not seem to show any difference in performance between Underweight and Overweight body stimuli across all Race Types.

The above findings suggested that perceptual decision of body size could be affected by many factors, and we have shown that gender and race type of the stimuli and the participants are critical features that should not be ignored. Importantly, we are not only interested in the accuracy rates, but the type of errors participants had made are equally vital, as their errors can inform us the direction of the perceptual bias relative to the standard definition of the BMI weight category.

### What kind of errors do people make?

#### Classification tree analysis reliably separated performance

**Figure 2:**
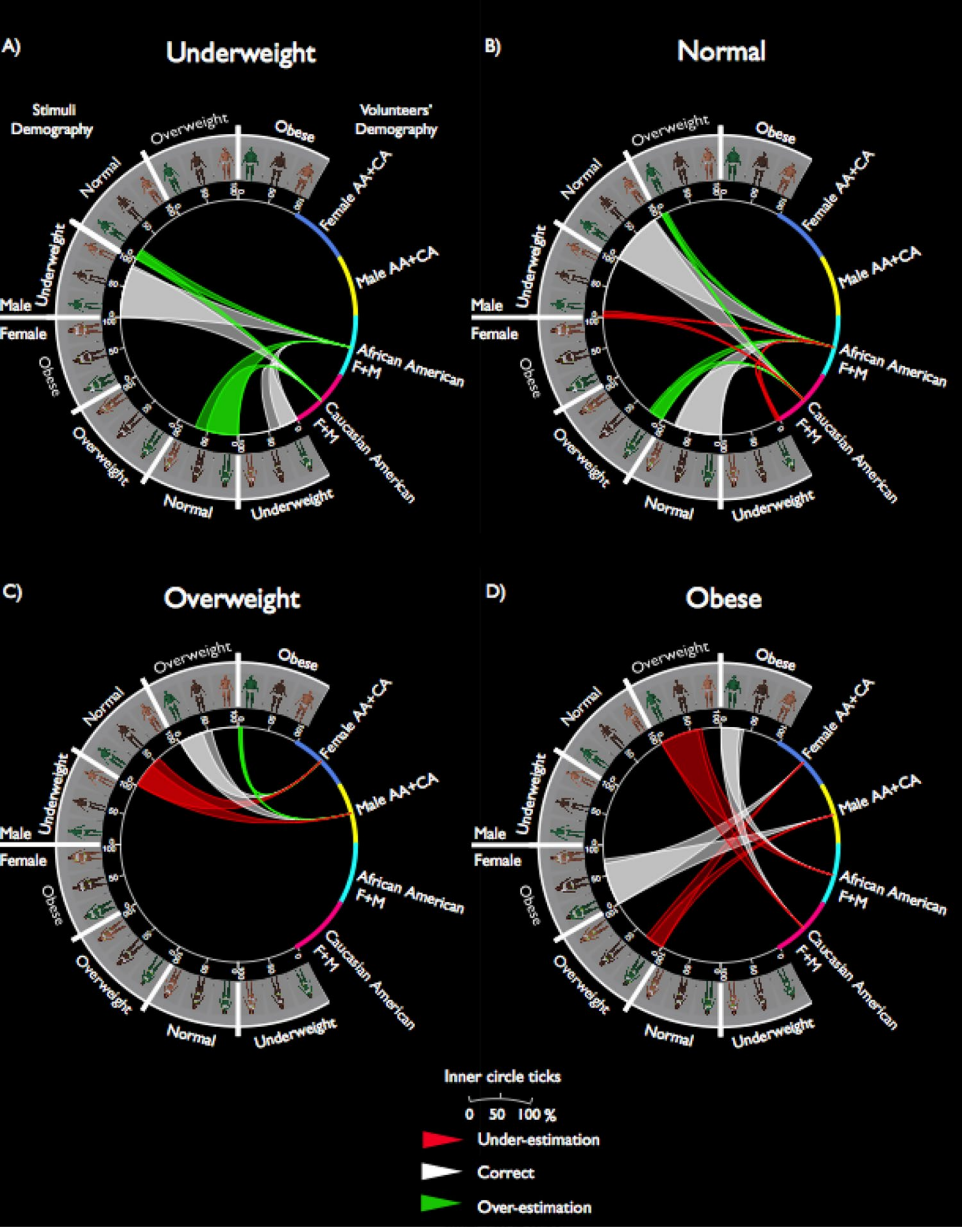
Circos^31^ plots are used to visualize significant nodes from the decision tree analysis for each weight categories (**A**) Underweight, (**B**) Normal, (**C**) Overweight, and (**D**) Obese. In each circos^31^ plot, the right side shows participants’ demography (F = female, M = male, AA = African American, CA = Caucasian-American), the rest of the circos^31^ marks stimuli demography and stimuli weight category. The width of the “triangle” illustrates the percentage of responses from participants. The inner circle ticks mark the percentage of responses; participants could have made three types of responses: correct estimation (white triangles), under-estimation (red triangles), or over-estimation (green triangles). Also see Supplementary Table 1 for a summary table of the decision tree results for each BMI category.

In order to capture the complex and hierarchical categorization patterns associated with correct- and mis-classification, we have categorized participants’ responses as correct estimation (coded as 0), under-estimation (coded as − 1), and over-estimation (coded as + 1), which allow the use of multi-variate analysis. A classification tree, a data-driven machine learning approach, was adopted—an algorithm that implements recursive partitioning of data into subgroups, at each step of the algorithm, where the data are divided to a single explanatory variable^30^. First, we have found a significant partitioning of the four BMI categories (*X*^2^(6, N = 21,525) = 8533.95, *p* < 0.0001, correct estimation CI = [0.60, 0.62], under-estimation CI [0.22, 0.25], over-estimation CI [0.13, 0.15]), suggesting that this data-driven approach can reliably parcellate responses into those expected groups. Specifically, our classification tree results showed that our participants did best with Normal stimuli, with 80.4% of correct responses, 14% of over-estimation responses, and 4.6% of under-estimation responses (*X*^2^(6, N = 5380) = 8533.96, *p* < 0.0001, correct estimation CI = [0.79 0.81], under-estimation CI [0.02, 0.07], over-estimation CI [0.12, 0.17]). This was followed by 61.5% of accurate responses for Underweight stimuli, with 38.4% over-estimation responses (*X*^2^(6, N = 5384) = 8533.96, *p* < 0.0001, correct estimation CI = [0.59, 0.63], over-estimation CI [0.36, 0.40]). Performance for Overweight stimuli was slightly worse than Underweight, with 59% of correct responses, 35.7% under-estimation, and 5% over-estimation responses (*X*^2^(6, N = 5379) = 8533.96, *p* < 0.0001, correct estimation CI = [0.57 0.60], under-estimation CI [0.33, 0.37], over-estimation CI [0.02, 0.07]). Performance was worse for Obese stimuli, with only 44% correct responses, but 56% of under-estimation responses (*X*^2^(6, N = 5382) = 8533.96, *p* < 0.0001, correct estimation CI = [0.46, 0.42], under-estimation CI [0.57, 0.54]). This pattern is of course consistent with the main effect of weight category with percent accuracy (see Fig. 2 and Supplemental Figure 2).

#### Underweight bodies: impact of the gender of the stimuli and race type of the participants

Overall, performance was better for male stimuli (85% accuracy) than for female stimuli (38.2%) (*X*^2^(1, N = 2692) = 1244.26, *p* < 0.0001) (Fig. 2A). When viewing female underweight bodies, African–American participants often over-estimated the female body weight (70.7% over-estimate vs 29.3% correct, CI [0.67, 0.73] [0.17, 0.26]), whereas Caucasian participants did not exhibit such bias (52.9% over-estimate vs 47.1% correct; CI [0.49, 0.56] [0.43, 0.50]; *X*^2^(1, N = 1349) = 90.427, *p* < 0.0001), demonstrating a significant difference in decision-making between African–American and Caucasian participants. In contrast, when viewing male underweight body stimuli, both African–American and Caucasian participants performed accurately and made fewer errors of mis-categorization, but Caucasians performed relatively better than African–American (CA 86.8% correct vs AA 83.1%; CI [0.84, 0.88] [0.80, 0.85]; *X*^2^(1, N = 1346) = 7.56, *p* < 0.0001). Our results suggested that while participants were accurate in categorizing male underweight bodies, African–American participants tended to over-estimate underweight female bodies, as they have consistently mis-categorized underweight female bodies as normal weight. Thus, this result showed a discrepancy between the perceptual weight boundaries and the standard BMI weight category.

#### Normal weight bodies: impact of the gender of the stimuli and race type of the participants

We were also able to identify biases associated with gender of the body stimuli and race type of the participants (Fig. 2B). Overall performance for normal weight stimuli was better for male stimuli than female stimuli (F: 76.1% vs M: 84.9%; CI [0.74, 0.77] [0.83, 0.86]; *X*^2^(2, N = 2693) = 313.619, *p* < 0.0001), participants made more over-estimation errors for female stimuli than for male stimuli (22.3% vs 7.5%, CI [0.18, 0.25] [0.03, 0.11]). Furthermore, for female stimuli, Caucasians performed slightly better than African–American participants, whereby AA made significantly more over-estimation errors (CA: 77.30% vs AA: 74.80%; CI [0.15, 0.24] [0.19, 0.29]), whilst CA made more under-estimation errors than AA (CA: 2.70% vs AA: 0.60%; CI [− 0.02, 0.07] [− 0.04, 0.05]; (*X*^2^(2, N = 1348) = 24.382, *p* < 0.0001)). In contrast, for male stimuli, responses were quite accurate relative to those for female stimuli. AA performed better than CA (AA: 85.90% vs CA: 83.90%, CI [0.83, 0.87] [0.81, 0.86]), but AA made more over-estimation errors than CA (AA: 9.20% vs CA: 5.80%, CI [0.04, 0.14] [0.006, 0.10]), whereas CA showed more under-estimation errors than AA (CA: 10.3% vs AA: 4.8% CI [0.05, 0.15] [− 0.003, 0.10]); *X*^2^(2, N = 1350) = 37.49, *p* < 0.0001). Overall, participants consistently slightly over-estimated normal female bodies as overweight, whereas they were quite accurate in categorizing males in the normal weight category. Once again, our data have suggested there is a misalignment between the perceptual and BMI boundaries.

#### Overweight bodies: impact of gender of the stimuli, race of the stimuli, and gender of the participants

**Figure 3:**
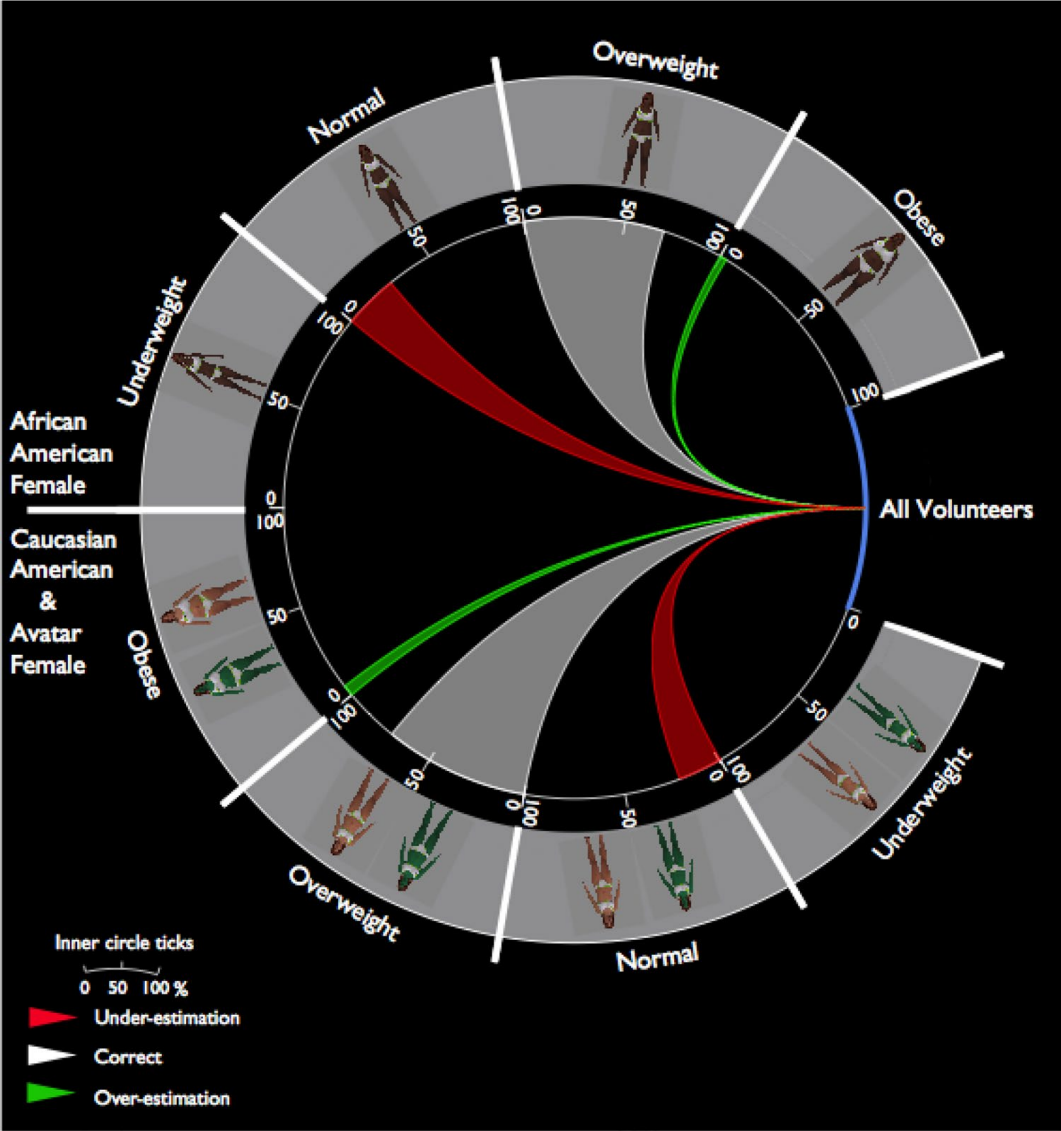
Categorization of Overweight Bodies. Circos^31^ plot illustrates the impact of gender and race type of the stimuli during categorization of overweight bodies. Specifically, for female stimuli, participants showed slightly but significantly higher accuracy rate for CA and Avatar bodies than for AA bodies, and they made more under-estimation errors for AA stimuli.

Overall performance for Overweight stimuli was significantly better for female stimuli than for male stimuli (F: 71.50% vs M: 47.10%; CI [0.69, 0.73] [0.44, 0.49]), participants made more under-estimation errors for male than for female stimuli (M: 47% vs F: 23.70%); CI [0.45, 0.50] [0.20, 0.27]; *X*^2^(2, N = 5379) = 352.55, *p* < 0.0001). This result is consistent with the significant interaction between the gender and weight categories of stimuli reported earlier (see Fig. 2C), where participants were more accurate for overweight female stimuli than for overweight male stimuli. Intriguingly, we also observed an impact of the gender of the stimuli and the participants (Fig. 2C), where for male stimuli, male participants performed significantly better than female participants (M: 54.70% vs F 39.50%; CI [0.51, 0.58] [0.35, 0.43]), and female participants showed more under under-estimation errors (F: 55.80% vs M: 39.60%; CI [0.52, 0.59] [0.35, 0.43]). An impact of gender and race type of the stimuli (Fig. 3) was also found; our data showed that for female stimuli, participants showed higher accuracy for CA and Avatar stimuli than for AA stimuli (CA + AV: 72.4% vs AA: 69.6%, CI [0.17, 0.26] [0.65, 0.73]), and they made more under-estimation errors for AA stimuli (*X*^2^(2, N = 1792) = 12.82, *p* < 0.005. These results illustrate that unlike categorization of underweight and normal female bodies, where participants tended to over-estimate, an opposite pattern was observed for overweight male bodies, participants tended to under-estimate. Hence, the misalignment between perception and BMI weight category depends on the gender of the stimuli and participants, as well as the race type of the stimuli.

#### Obese bodies: impact of gender of the stimuli, gender of the participants, and race of the participants

Consistent with the accuracy results above, performance (Fig. 2D) was lowest for the Obese stimuli (44%) and there was also an impact of stimuli gender, where performance was better for female stimuli than male stimuli (F: 63.2% vs M: 24.9%; CI [0.60, 0.65] [0.21, 0.28]; (*X*^2^(1, N = 2690) = 801.23, *p* < 0.0001). We also observed an impact of gender and race of the participants. Specifically, we found a significant difference between male and female participants, where male participants (66.90%; CI [0.63, 0.69]) showed more accurate performance for female Obese stimuli relative to female participants (59.50%; CI [0.56, 0.62]) (*X*^2^(1, N = 1346) = 15.587, *p* < 0.0001). Intriguingly, for obese male stimuli, AA participants performed worse than CA, whereby they made more under-estimation errors for Obese bodies (AA: 21.10% vs CA: 28.60; CI [0.16, 0.25] [0.24, 0.33]; (*X*^2^(1, N = 1348) = 20.272, *p* < 0.0001). Similar to the performance for Overweight stimuli, participants tended to under-estimate male Obese bodies, but they were relatively accurate for female Obese bodies.

#### Participants’ BMI

Due to the heterogeneity in BMIs among participants and small sample size, we did not test the impact of participants BMI group on body size discrimination performance. Nonetheless, each participant’s BMI was calculated (weight divided by the square of the body height), and a between-subject (participants’ gender and race as factors) univariate ANOVA was performed. This showed that there was a significant main effect of participants’ ethnicity (*F*(1,56) = 12.14, *p* < 0.001). No other effects were observed. Follow-up independent sample t-tests illustrated that there was a significant difference in BMIs between AA and CA participants (*t* = 3.51, *p* < 0.001), suggesting that AA participants (female: M = 28.46, SD = 4.54; male: M = 29.30, SD = 5.59) had significantly higher BMIs than CA participants (female: M = 24.40, SD = 3.71; male: M = 25.82, SD = 2.11). The average BMI within AA participants was in the Overweight category, while for CA, their BMIs were between the Normal and Overweight categories. Putting the above results in the context of accuracy performance, since no significant effects were related to the race type of the participants, it is unlikely that participants’ own BMIs affect accuracy performance in size estimation of others in our current experiment. As for the effects reported in the decision tree analysis, a couple of significant effects concerning the race type of the participants and the gender of the stimuli were found. Specifically, AA participants frequently over-estimated the female Underweight bodies, whilst CA participants did not exhibit such bias. Furthermore, AA participants also performed worse than CA in Obese male stimuli, by making more under-estimation errors. It remains unclear the extent to which participants’ BMIs affect body size estimation of others in our current study; our results have once again underscored the complex relationship between attributes from the participants and the stimuli, highlighting the importance of using a diverse approach in body size research.

## Discussion

By providing corroborating evidence from univariate and multi-variate analyses to investigate body size perception, we are able to identify the complex relationship between gender and race types of the stimuli and of the participants, and the impact of these factors on body size categorization. In particular, we have revealed that performance (percent accuracy) for body stimuli is not uniform, whereby participants performed best for Normal weight and worst for Obese stimuli. We also revealed evidence of an interaction between weight category and gender of the stimuli; participants were more accurate for Underweight and Normal male stimuli (leaner size) relative to the same weight category of female bodies, but they were more accurate for overweight and obese female stimuli compared to male bodies of the same size.

Multi-variate decision tree analysis provided not only consistent results but has pinpointed the direction of estimation errors. Specifically, it revealed that while our participants were reliably making more under-estimation errors for Overweight and Obese male stimuli, they were quite accurate when categorizing Overweight and Obese female stimuli. The overall decision tree pattern (Fig. 2 and Supplemental Figure 2) suggests a strong bias for male stimuli where participants showed an increasing number of errors from leaner to bigger bodies, particularly under-estimation errors. Importantly, there was an expansion of Normal weight category, such that for male stimuli, while a high percent accuracy was found for Normal weight (Fig. 2B), there were also substantial under-estimating errors for identification of Overweight male bodies (Fig. 2C), where participants consistently mis-identified Overweight as Normal weight. Thus, it ndicated that the perceptual BMI for Normal male bodies is now higher than the recognized BMI. For female stimuli however, an expanded boundary for Normal size was found in the opposite direction. Participants have categorized Underweight as Normal (Fig. 2A) as well as accurately categorized Normal bodies (Fig. 2B), suggesting that the averaged perceptual BMI for ormal female bodies is now lower than the recognized BMI. The fact that both male and female participants shared many of the same biases also suggests that visual learning plays a critical role in developing these specific biases. Part of these results was consistent with previous findings; for example, it has been reported^5,6^ that participants (predominately Caucasian women) make more under-estimation errors when judging Caucasian Overweight or Obese male bodies. It is also worth highlighting that due to the diverse range of our stimuli and equal sampling across both gender and race of participants in the current study, we are able to capture opposite response patterns when judging male vs female bodies, thus, a more complex pattern than previously reported.

As mentioned earlier, prior work has primarily reported perceptual biases for own body weight that might associate with race or gender types, while others have reported biases when judging others’ bodies, but theyhave primarily focused on a particular gender, race, and/or weight category of the stimuli or participants. Our current study focused on assessing others’ body weight as observers, accounting for race and gender of both stimuli and participants. We found that perceptual errors could be associated with characteristics of the participants and the stimuli. For example, all participants, regardless of their race and gender, showed more under-estimating errors by mis-categorizing AA female overweight bodies as normal (Fig. 3). Intriguingly, (Fig. 2A) when judging female underweight bodies (all race types), AA participants (both genders), showed a stronger over-estimating bias for female underweight bodies, mis-categorizing those images as normal weight. CA participants, however, did not exhibit such bias.

It has been well-established in the face perception literature that people are more accurate in recognizing and identifying faces of their own race compared to other race groups. This discrepancy in performance is known as the “*other-race effect*”^32–40^. In the context of body weight perception, our current results did not show any other-race effect; no interaction between stimulus and participants’ race types was found in the univariate analysis or multi-variate analysis. However, we identified various participant-specific race effects from the multi-variate results. For example, we found that AA participants were slightly better at categorizing Obese male bodies relative to CA participants. AA participants also over-estimated Underweight female bodies, as they had consistently mis-categorized Underweight female bodies as Normal size (and this effect was not present in CA participants). For normal male bodies, AA performed better than CA, but AA made more over-estimation errors than CA. Interestingly, some stimuli-specific race effects were also identified. Overall, categorization performance was slightly (but significantly) better for Avatar stimuli in terms of percent accuracy. Multi-variate analysis further revealed that during categorization of female overweight stimuli, participants showed higher accuracy for Avatar and CA bodies than for AA (Fig. 3). A recent study^20^ had used visual adaptation to study the after-effect following repeated exposure of Asian or Caucasian female bodies, and their results seemed to be consistent to our findings. They also reported a lack of “other-race effect” at the stimuli level, but they reported that Asian participants seemed to show a weaker adaptation effect relative to Caucasians; however, the effect was not specific to Asian or Caucasian stimuli.

*“Own-gender biases”* have also been reported in face perception literature^41^. People are better at recalling or recognizing faces of their own gender relative to faces of the opposite gender^41,42^. Limited work has been conducted regarding gender-biases in body perception. Our recent study^43^ investigated gaze-pattern during perception of upright vs inverted bodies, but observed no differences in eye-movement patterns between male and female participants during a same/different categorization task of male body images. Multi-variate analysis in the current study has identified significant differences in performance between viewing female and male body images. There is a stimuli- and participant-specific gender effect that is particularly prominent for Obese bodies. Specifically, a marked difference was found between male and female participants, where male participants showed significantly higher accuracy for female Obese bodies. For male Overweight bodies, male participants performed better than female, while female participants showed more under-estimation errors. This suggests that under-estimation bias for Overweight male bodies was primarily driven by female participants. A stimuli-specific gender effect was also observed whereby, consistent with the univariate analysis, participants performed more accurately for Underweight male bodies than female Underweight bodies. Overall performance for Normal weight images was also better for male than female bodies. For Obese bodies, performance was better for female bodies, and there were also more under-estimation errors for male Obese bodies. These findings demonstrated that, by increasing the diversity in the stimuli and participants tested and by adopting a multi-variate approach, a more complex categorization pattern can be revealed. Furthermore, our observations of behavioural biases for higher BMI male stimuli and for lower BMI female stimuli seem to be consistent with the idea that partial overlapping or multiple gender-specific neural mechanisms may be at play during body size perception^24,25^.

Two major theories have been adopted to elucidate perceptual weight biases: the Weber’s law and contraction bias^12,13,44^. Specifically, the Weber’s law would predict that since detection of change of one’s body size is in constant proportion with one’s own weight, it is more diffcult to notice the change when one is overweight/obese. Alternatively, contradiction bias predicts that one’s perceived own BMI is inversely correlated with their own actual BMI. It has been reported that such correlation was only found during size estimation of participants’ own avatar, but did not generalize to estimating others’ body size^12^. While these theories may be helpful for explaining error in estimating one’s own weight, it is rather difficult to apply them to explain errors/biases during identification of others’ weight, especially when there are a lot more variables (race, gender, body weight, etc.) when dealing with “other bodies”. As we have shown here, estimation accuracies and errors interact with the type of stimuli presented in the experiment, thus illustrating that with increasing diversity in the stimuli, it might not be possible to show an “one-to-one mapping” using the above theories, as estimation decisions might be more complex than previously thought. While it is important to recognize that people have different body sizes, shapes, and other physical characteristics^19^, and that even BMI cut-off points may not capture variations in physiological measurements across cultures^45^, our current approach aims demonstrated that it is possible to capture and quantify some of the multi-dimensional visual characteristics, and it is critical that future work should also harness similar approaches.

Our findings here certainly do not attempt to capture categorization patterns for all types of bodies, and despite the constraints in our well-controlled paradigm (in real life, people with the same BMI may have different body shapes, and we see bodies from many different viewpoints other than straight-on), we have taken an important first step to quantify complex patterns in body weight perception. Finally, we believe that providing a careful characterization of perceptual biases in body weight here may lead to better diagnostic decision-making and development of personalized intervention programmes in both clinical and non-clinical settings.

## Materials, methods and procedures

### Participants

Sixty participants were recruited for this study (age 20–44). Specifically, four groups of participants were tested, 15 participants per group with equal numbers of female and male participants, equal numbers of participants who had identified themselves as African American and Caucasian American. The current protocol was approved by the Institutional Review Board of the University of Tennessee Health Science Center (Protocol no. #15-03683-XP). The current research had been performed in accordance with the Declaration of Helsinki. All participants gave written informed consent and were compensated for their participation.

### Design and materials

**Figure 4:**
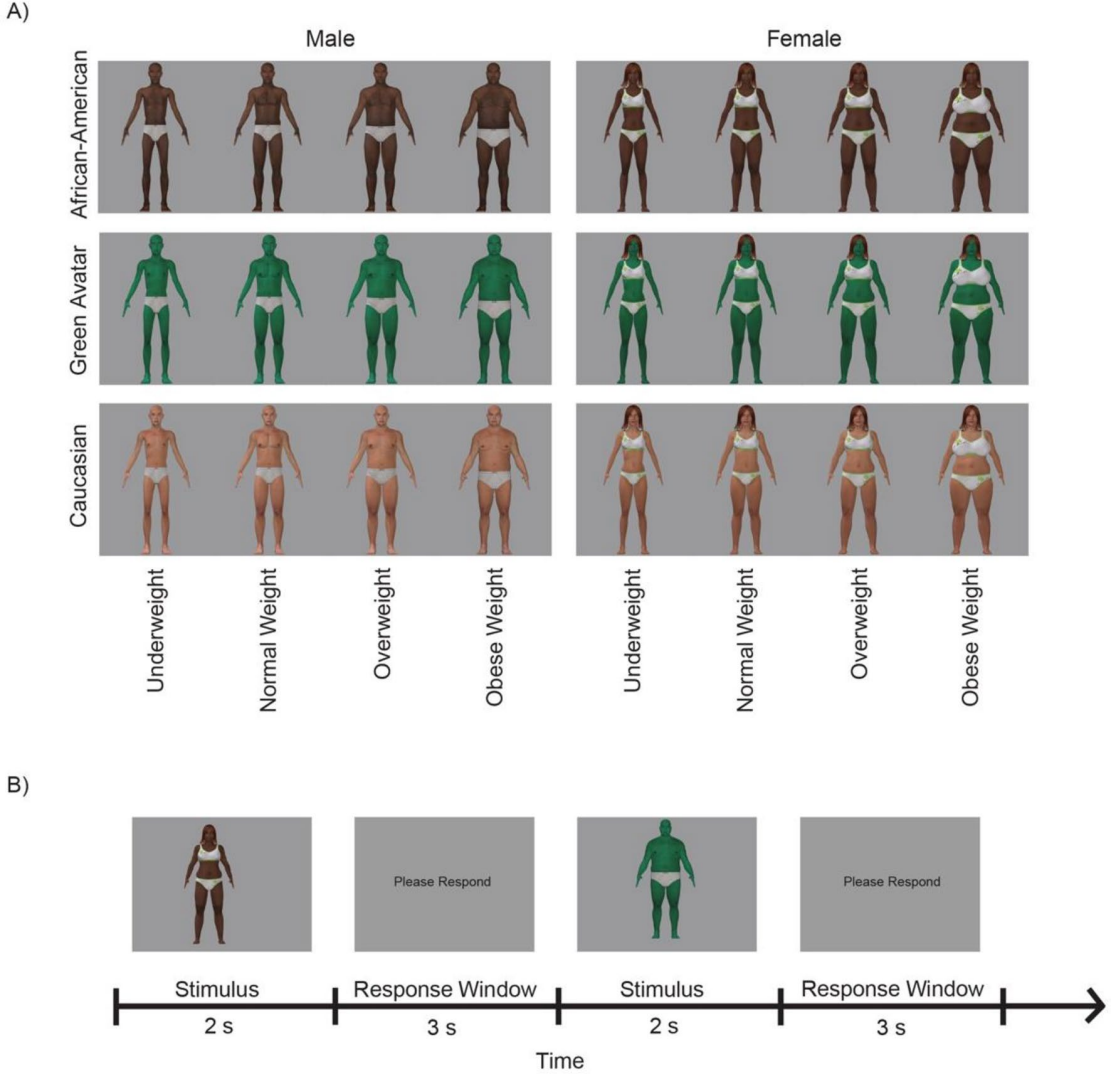
Experimental paradigm for the visual categorization task. (**A**) Example stimuli set, a condition-rich paradigm consisting of a total of 24 conditions through a 2 × 3 × 4 within-subject factorial design, comprised of 2 gender types (male,  female), 3 race  types (African–American, Caucasian, Green Avatar as a control type), and 4 types of BMI weight categories (underweight, normal, overweight, obese) of the body stimuli. Each condition consisted of 5 computer-generated individual identities. (**B**) Example trial, where each body stimulus was presented for 2 s, followed by a 3-s response window. Participants were asked to judge whether each stimulus was underweight, normal, overweight, or obese by pressing the corresponding button on the keyboard.

A psychophysical experiment (visual body categorization) was carefully designed to study the effects of demographic features by presenting 120 body stimuli × 3 runs, resulting in a total of 360 body stimuli viewed by each participant , with each run lasting for approximately 10 min. This experiment was a rich presentation paradigm consisting of 24 conditions, a 2 × 3 × 4 within-subject factorial design. This was comprised of 2 Gender body stimuli (male, female), 3 Race of body stimuli (African–American/AA, Caucasian American/CA, Green Avatar as a control race), and 4 types of BMI weight categories of the body stimuli (Underweight, Normal, Overweight, Obese; see Fig. 1). Each condition consisted of 5 computer-generated individual identities (see Fig. 4A). These stimuli were computer-generated images, polygon meshes (Dyna Models) created by Pons-Moll and colleagues^46^ using 4D cameras to capture images of actors with a range of BMI (from underweight to obese). These meshes were exported to Poser Pro (Smith Micro Software 2014), where they were then customized in order to generate additional race types and identities within each BMI category. We then performed width transformation in Poser Pro by 0%, $\pm$ 5% or $\pm$ 10%. Also see Supplementary Figure 1 for body stimuli weight categories and the standard BMI boundaries.

The experiment was presented to the participants on a computer screen, one stimulus at a time. This was an event-related design, where stimuli and position of the stimuli were randomized using Optseq2^47^, and the position of each image was slightly jittered from the center of the screen to minimize estimation using low-level visual differences between images. Participants were asked to estimate if the body is underweight, normal, overweight, or obese by pressing the corresponding key. Both accuracy and error rates were measured (Fig. 4B).

### Analysis

#### Accuracy

We measured the correct responses and calculated percent accuracy for each condition and for each participant. Percent accuracy was then submitted to a repeated measures factorial ANOVA, with the Stimulus Gender (Female, Male), Stimulus Race Types (African American, Caucasian American, Green Avatar), and Stimulus Weight Categories (Underweight, Normal, Overweight, Obese) as within-subject factors, and Participant Genders (Female, Male) and Participant Race Types (African American, Caucasian American) as between-subject factors.

#### Participants’ BMI

Each participant’s weight and height were recorded and their BMI values were subsequently calculated within each group.

#### Classification tree analysis

To gauge the decision-making patterns, we had not only measured the accuracy rates (correct-estimation) but also the direction of biases (under-estimation, over-estimation of the weight categories). Again, we had categorized participants’ responses as correct estimation (coded as 0), under-estimation (coded as − 1), and over-estimation (coded as + 1) for this analysis. To expose the specific observant and stimuli profiles leading to correct classification, under-estimation, or over-estimation, we implemented a classification tree analysis using a Chi-Squared Automatic Interaction Detector (CHAID) algorithm. The CHAID algorithm splits parent nodes into children nodes using the predictor yielding the minimum p-value by chi-squared test that is lower than the splitting criteria (0.05 in our case). CHAID uses Bonferroni-adjusted p-values since the selection of the predictor with the smallest p-value is a multiple testing task. The algorithm is terminated when there is no Bonferroni-adjusted p-value lower than the determined significance level. In addition, we also set the minimum size of parent nodes to 50, the minimum size of children nodes to 25, and maximum depth (max number of splits) to 3 ^30^. As a non-parametric classification method, the main concern about classification trees is over-fitting, leading to a lack of generalizability of the model. To control overfitting, we implemented fivefold cross-validation. In fivefold cross-validation, the cohort was divided into five equal-size subgroups. Next, a tree was developed through a combination of 4 subgroups (comprising 80% of the original sample) and tested on the remaining one subgroup (20%). The associated risk for each case in the test data was calculated for each of the 5 subgroups; the average of the risk across the 5 test samples were presented as the cross-validation risk. Smaller values of cross-validation risk indicate that the produced classification model is generalizable. The final tree represented was the one built on the full cohort.

### Ethical approval

This study was approved by University of Tennessee Health Science Center Institutional Review Board. Protocol no. #15-03683-XP.

## Supplementary Information

Supplementary Information 1.

Supplementary Information 2.

## Data Availability

Datasets reported in the manuscript will be shared via the Brunel University London research repository—Figshare, reserved https://doi.org/10.17633/rd.brunel.11791461.
