# Supplementary material for: Misalignment between perceptual boundaries and weight categories reflects a new normal for body size perception
Source: Sci Rep. 2021 May 17;11:10442. doi: 10.1038/s41598-021-89533-5 (PMC8129102; doi:10.1038/s41598-021-89533-5)
Supplement: Supplementary file 1 — Supplementary Information 1. [file 41598_2021_89533_MOESM1_ESM.pdf]

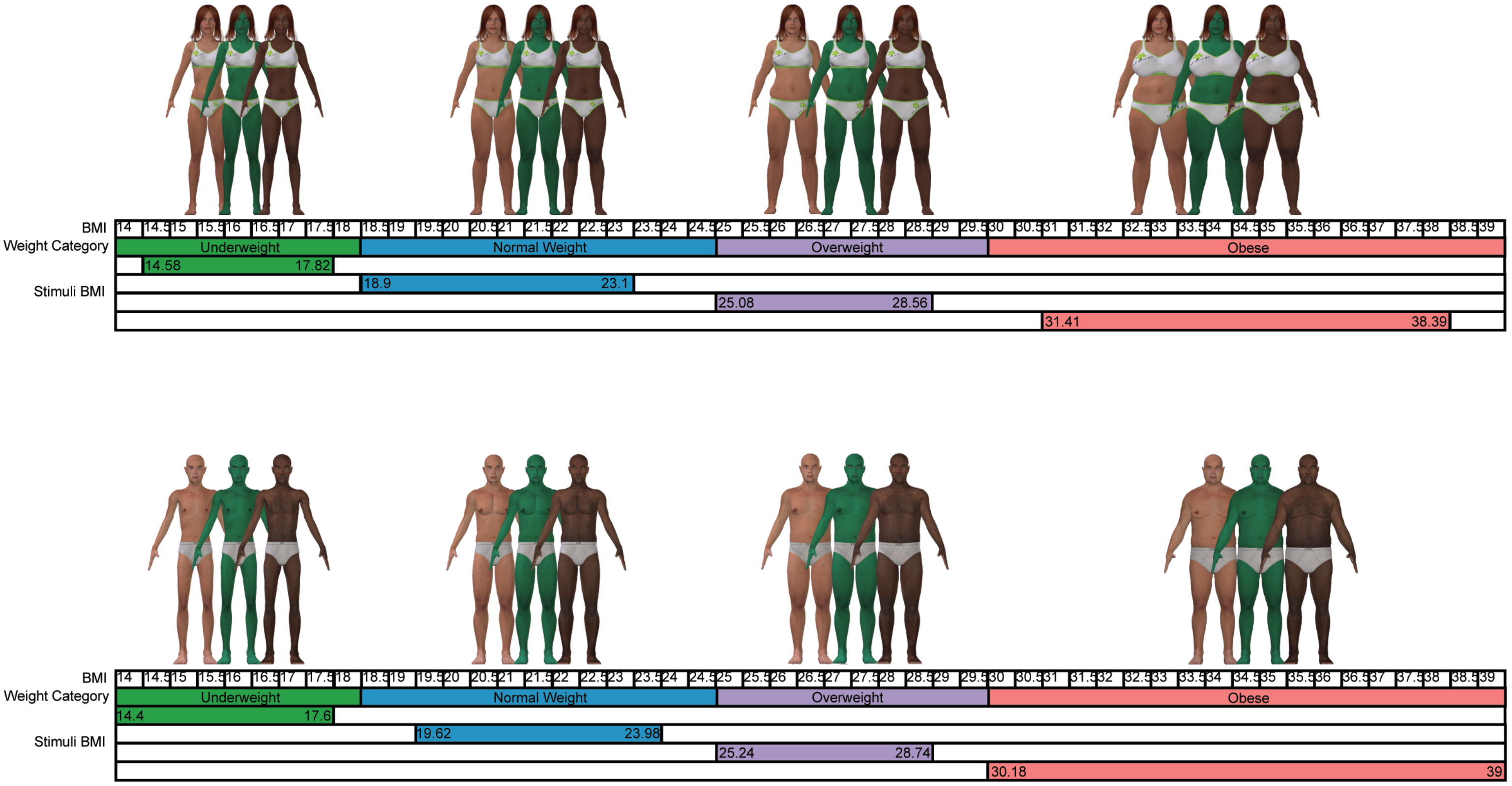

Supplementary figure 1: Female and male standard BMI boundaries and weight categories (underweight, normal, overweight and obese) along with the stimulus' BMI range.

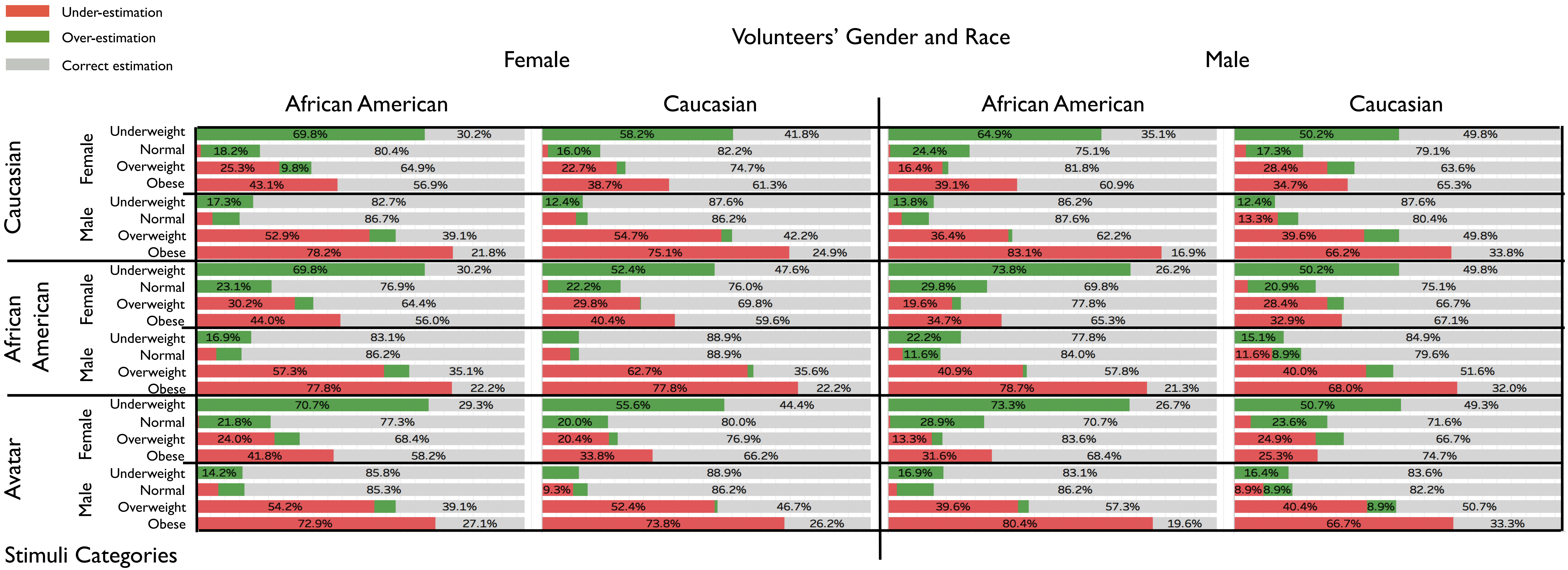

Supplementary figure 2: A summary table showing both percent accuracy (grey bars for correct estimation) and errors (green bars for over-estimation, red bars for under-estimation) for each condition (24 conditions) and each group of volunteers (4 groups).
