# Supplementary material for: Misalignment between perceptual boundaries and weight categories reflects a new normal for body size perception
Source: Sci Rep. 2021 May 17;11:10442. doi: 10.1038/s41598-021-89533-5 (PMC8129102; doi:10.1038/s41598-021-89533-5)
Supplement: Supplementary file 2 — Supplementary Information 2. [file 41598_2021_89533_MOESM2_ESM.pdf]

Supplementary table 1: A summary table of the decision tree results for each BMI category (> means higher accuracy; significant findings only)

Participants	***Stimuli***	***Stats***	***P values***
Everyone	Underweight Male > Female	$X^2(1, N = 2692) = 1244.26$	$p < 0.0001$
Caucasian > African-American	Underweight Female	$X^2(1, N = 1349) = 90.42$	$p < 0.0001$
Caucasian > African-American	Underweight Male	$X^2(1, N = 1346) = 7.56$	$p < 0.0001$
Everyone	Normal Male > Female	$X^2(2, N = 2693) = 313.61$	$p < 0.0001$
Caucasian > African-American	Normal Female	$X^2(2, N = 1348) = 24.38$	$p < 0.0001$
African-American > Caucasian	Normal Male	$X^2(2, N = 1350) = 37.49$	$p < 0.0001$
Everyone	Overweight Female > Male	$X^2(2, N = 5379) = 352.55$	$p < 0.0001$
Male > Female	Overweight Male > Female	$X^2(2, N = 1347) = 71.35$	$p < 0.0001$
Everyone	Overweight Caucasian & Avatar > African-American	$X^2(2, N =1792) = 12.82$	$p < 0.005$
Everyone	Obese Female > Male	$X^2(1, N = 2690) = 801.23$	$p < 0.0001$
Male > Female	Obese Female	$X^2(1, N = 1346) = 15.58$	$p < 0.0001$
Caucasian > African-American	Obese Male	$X^2(1, N = 1348) = 20.27$	$p < 0.0001$
